# Effect of non-steroidal anti-inflammatory drugs on colon carcinoma Caco-2 cell responsiveness to topoisomerase inhibitor drugs

**DOI:** 10.1038/sj.bjc.6600289

**Published:** 2002-05-06

**Authors:** P Ricchi, T Di Matola, G Ruggiero, D Zanzi, A Apicella, A di Palma, M Pensabene, S Pignata, R Zarrilli, A M Acquaviva

**Affiliations:** Dipartimento di Biologia e Patologia Cellulare e Molecolare ‘L. Califano’, Istituto di Endocrinologia ed Oncologia Sperimentale ‘G. Salvatore’ del Consiglio Nazionale delle Ricerche, Università ‘Federico II’, Napoli, 80131 Italy; Divisione di Oncologia Medica B, Istituto Nazionale Tumori, Fondazione G. Pascale, Napoli, Italy

**Keywords:** aspirin, NS-398, cell cycle, Bcl-2, colon cancer, topoisomerase inhibitors

## Abstract

Numerous studies demonstrate that the chemopreventive effect of non-steroidal anti-inflammatory drugs on colon cancer is mediated through inhibition of cell growth and induction of apoptosis. For these effects non-steroidal anti-inflammatory drugs have been recently employed as sensitising agents in chemotherapy. We have shown previously that treatments with aspirin and NS-398, a cyclo-oxygenase-2 selective inhibitor, affect proliferation, differentiation and apoptosis of the human colon adenocarcinoma Caco-2 cells. In the present study, we have evaluated the effects of aspirin and NS-398 non-steroidal anti-inflammatory drugs on sensitivity of Caco-2 cells to irinotecan (CPT 11) and etoposide (Vp-16) topoisomerase poisons. We find that aspirin co-treatment is able to prevent anticancer drug-induced toxicity, whereas NS-398 co-treatment poorly affects anticancer drug-induced apoptosis. These effects correlate with the different ability of aspirin and NS-398 to interfere with cell cycle during anticancer drug co-treatment. Furthermore, aspirin treatment is associated with an increase in bcl-2 expression, which persists in the presence of the anticancer drugs. Our data indicate that aspirin, but not NS-398, determines a cell cycle arrest associated with death suppression. This provides a plausible mechanism for the inhibition of apoptosis and increase in survival observed in anticancer drug and aspirin co-treatment.

*British Journal of Cancer* (2002) **86**, 1501–1509. DOI: 10.1038/sj/bjc/6600289 www.bjcancer.com

© 2002 Cancer Research UK

## 

Numerous studies have shown that the chemopreventive effect of Non-Steroidal Anti-inflammatory drugs (NSAIDs) on colon cancer is mediated through inhibition of cell growth and induction of apoptosis (Lu *et al*, 1995; Piazza *et al*, 1995; Shiff *et al*, 1995; Arber *et al*, 1997). In particular, several investigators have found that aspirin and other NSAID treatments correlate with a decrease in cell replication and with an increased proportion of cells arrested at the G0/G1 phase of the cell cycle (Piazza *et al*, 1995; Shiff *et al*, 1995, 1996; Arber *et al*, 1997). The molecular mechanisms responsible for the effect of NSAIDs on cell growth and apoptosis are controversial, being either related to their ability to inhibit cyclooxygenase activity and prostaglandin synthesis or independent from a prostaglandin pathway (Chan *et al*, 1998; Sheng *et al*, 1998; Smith *et al*, 2000).

More recently, several reports demonstrated that combinations of different agents for cancer prevention is more effective than using either a single agent alone (Li *et al*, 1999; Torrance *et al*, 2000). Combination therapy is a strategy currently used to treat cancer and NSAIDs, for their effects on cell growth and apoptosis, are potential chemotherapeutic agents to be used alone or in combination with conventional anticancer drugs. Only a few studies have accurately evaluated this possibility (Lundholm *et al*, 1994; Sinicrope *et al*, 1996; Duffy *et al*, 1998) and more recently cyclooxygenase-2 (Cox-2) selective inhibitors have been found to enhance the sensitivity to anticancer drugs (Soriano *et al*, 1999; Hida *et al*, 2000). Thus, the need for an accurate evaluation of all the potential interactions between these two classes of drugs.

In a previous work we have shown that aspirin treatment may affect the proliferation, differentiation and apoptosis of the human colon adenocarcinoma Caco-2 cells; in particular, we observed that, depending on the doses, aspirin treatment may induce apoptosis and a significant DNA synthesis inhibition associated with a modification in the level of the insulin-like growth factor II (IGF-II) (Ricchi *et al*, 1997), an autocrine growth factor for this cell line (Zarrilli *et al*, 1994, 1996). More recently, we have demonstrated that NS-398, a Cox-2 selective inhibitor, also affects Caco-2 DNA synthesis and apoptosis (Di Popolo *et al*, 2000).

Accumulating evidence suggest that interference with cell cycle and/or with the intracellular growth factor (receptor)-activated signal transduction pathways are key modulators of cellular response to chemotherapeutic agents (Donaldson *et al*, 1994; Ciardiello *et al*, 1996; Tortora *et al*, 1997; Chen *et al*, 1997; De Luca *et al*, 1997; Lin *et al*, 1998). Because aspirin and NS-398 are able to inhibit cell growth, we asked whether they might modify sensitivity to anticancer drugs. Because cell killing induced by the topoisomerase poisons irinotecan (CPT 11) and etoposide (VP-16) is strictly cell cycle dependent (Nitiss and Beck, 1996; Goldwasser *et al*, 1995) and ultimately activates a pathway of programmed cell death, we have decided to use these anticancer drugs to evaluate our hypothesis. Irinotecan (CPT 11) was selected also because it is one of the most active anticancer agent in colon cancer (Rougier *et al*, 1998). We have additionally evaluated whether the effect of aspirin and NS-398 treatments on the sensitivity of Caco-2 cells to anticancer drugs correlate with any modification in the level of the bcl-2 family protein, one of the key regulator factors in apoptosis (Yang *et al*, 1995; Korsmeyer 1999).

Here we find that, depending on the concentration, NS-398 treatment poorly affects Caco-2 anticancer sensitivity, while aspirin treatment, counteracting anticancer drug-induced cell cycle modification and inducing the expression of bcl-2, is able to substantially alter Caco-2 anticancer drug-induced apoptosis and overall viability.

## MATERIALS AND METHODS

### Cell growth and culture

Caco-2 cells were routinely grown in 100 mm plastic dishes at 37°C in a humidified incubator 5% CO_2_-95% air atmosphere in Dulbecco's Modified Eagle's Medium (DMEM) supplemented with 10% foetal calf serum, glutamine (2 mM), penicillin (100 u ml^−1^), and streptomycin (100 μg ml^−1^) and buffered with N-2-hydroxy-ethylpiperazine-N′-2-ethane sulphonic acid (HEPES) (20 mM). Caco-2 cells were seeded at 5 × 10^4^ cells ml^−1^ and were routinely sub-cultured when about 80% confluent. The culture medium was changed every other day. Confluence was reached 6–8 days after the inoculum and the stationary phase on day 10. Cell cultures were stopped after 15 passages.

Etoposide and irinotecan were a gift from Bristol-Myers-Squibb (Rome, Italy) and Rhone Poulen, respectively; anticancer drugs were diluted in DMEM to prepare 500× concentrated solutions. Aspirin (Sigma, Milan, Italy) was dissolved in a 0.1 M Tris-HCl pH 7.8 solution. The solutions were buffered with Tris base to obtain the final pH equal to that of control DMEM medium and prepared every two weeks. Experiments were performed in the absence or in the presence of aspirin. In aspirin untreated cells (control) anticancer drugs were added for 48 h at day 4 of culture. In treated cells aspirin was added at day 3 of culture for 24 h. Anticancer drugs were added for 48 h in presence of aspirin or NS 398 (co-treatment) without changing media.

### Cell cycle analysis and apoptosis detection

In order to define the cell cycle distribution and apoptosis rate, Caco-2 cells were trypsinised, pelletted, fixed and Propidium Iodide (PI) stained as previously described (Nicoletti *et al*, 1991). PI staining fluorescence of individual cells was analysed by using a FACS Calibur flow cytometer apparatus (Becton & Dickinson, Mountain View, CA, USA) and the MODFIT analysis software. For each sample, at least 20 000 events were stored.

Apoptosis/necrosis ratio was additionally evaluated by using annexin V-FITC/PI double staining technique. Briefly, trypsinized Caco-2 cells were collected, including floating apoptotic cells and the cells spontaneously detached during washing procedure, and annexin V-FITC and PI co-stained by using a detection kit from Medical & Biological Laboratories Co, Ltd, Naka-ku Nagoya Japan, according to the manufacturer's instructions. Fluorescence analysis was performed by a flow cytometer apparatus (Becton & Dickinson, Mountain View, CA, USA) and the Cell Quest analysis software. For each sample, at least 30 000 events were stored. Quadrant settings were based on the negative control. Each experiment was repeated at least three times.

### Plating efficiency assay

To determine anticancer drug responsiveness of Caco-2 cells a plating efficiency assay was performed following drug removal. After each treatment, cells were trypsinised, washed and seeded (15 000 cells × well) in triplicate in 24 multiwell cluster dishes and counted at days 1, 4, 6, 8 and 11 of culture.

### Western blot analysis

Mouse monoclonal antibody to Bcl-2 was from Santa Cruz Biotechnology, Santa Cruz CA, USA. Cells were washed in cold PBS and lysed for 10 min at 4°C with 1 ml of lysis buffer (50 mM Tris, pH 7.4, 0.5% NP40, 0.01% SDS) containing protease inhibitors. Lysates from adherent cells collected by scraping were centrifuged at 12 000 *g* for 15 min at 4°C. The protein concentration in cell lysates was determined by Bio-Rad Protein Assay (Bio-Rad, Richmond CA, USA) and 50 μg of total protein from each sample was analysed. Proteins were separated by a 12% SDS-polyacrylamide gel electrophoresis and transferred on nitro-cellulose membrane (Hybond-ECL Nitrocellulose, Amsherman, Rainham, UK). Membranes were blocked in 5% non-fat dry milk, and after three washes, were incubated for 1 h at 4°C with 0.5 μg ml^−1^ of mouse monoclonal primary antibody in PBS. After five washes, filters were incubated for 1 h at 4°C with horseradish peroxidase-conjugated anti-mouse secondary antibodies (Bio Rad) diluted 1 : 2000 in PBS, 0.2% Tween. The membranes were then washed and protein bands were detected by an enhanced chemiluminescence system (Amersham Pharmacia Biotech). Control for loading and transfer was obtained by probing with anti-α-tubulin (Sigma) at 1 : 4000 dilution. For quantitation of immunoblots, relative intensities of bands were quantified by densitometry with a desk scanner (Pharmacia Discovery system) and RFLPrint software (PDI, New York, USA).

### Statistical analysis

Statistical comparisons were performed using the Mann–Whitney *U*-test. A probability value *P*<0.05 was considered a significant difference.

## RESULTS

### Effect of aspirin treatment on proliferation and apoptosis of Caco-2 cells

Our previous data indicated that a 24 h aspirin treatment on Caco-2 cells at doses ranging from 1 to 10 mM determined a dose-dependent inhibition of DNA synthesis and a significant increase in levels of apoptosis (Ricchi *et al*, 1997). In the present work, we confirmed these results on cell growth for a 72 h treatment (data not shown) and evaluated again the effects on apoptosis by analysing the percentage of sub-G1 population at flow cytometry. Treatment with aspirin for 72 h, caused a dose-dependent increase in apoptosis starting from the concentration of 5 mM aspirin; the percentage of apoptotic cells was approximately 6% in control cells and 5, 9 and 12% in cells treated with aspirin at 2, 5 and 10 mM, respectively (data not shown).

The above data suggest that aspirin acts in Caco-2 cells as anticancer agent with cytostatic properties and ability to induce apoptosis at high dosages. Therefore we decided to select these antiproliferative, apoptotic dosages of drugs to study whether they could modify Caco-2 sensitivity to anticancer drug treatments.

### Effect of aspirin co-treatment on CPT 11 and Vp-16-induced apoptosis

**Figure 1:**
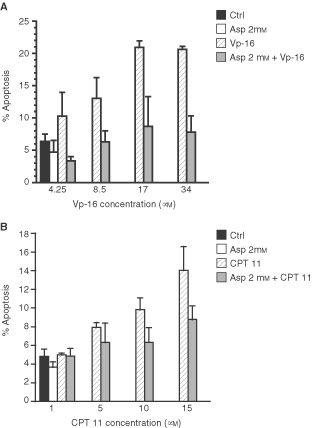
Effect of 2 mM aspirin co-treatment on VP-16-(**A**) and CPT 11(**B**)-induced apoptosis in Caco-2 cells. Cells at day 3 were incubated with aspirin for 24 h, then anticancer drugs at the indicated concentrations were added in presence of 2 mM aspirin for 48 h. Apoptosis was calculated as the percentage of cells showing a sub-diploid DNA peak as described in Materials and Methods. Data are expressed as mean±s.d.

To determine the effect of anticancer drugs on apoptosis, Caco-2 cells were exposed for 48 h at different concentrations of VP-16 and CPT 11 and DNA ploidy was analysed by flow cytometry. The effects on apoptosis in Caco-2 cells are shown in Figure 1

. Both Vp-16 and CPT 11 dose-dependently increased the levels of apoptosis in Caco-2 cells: Vp-16 increased apoptosis from 8% at 4.25 μM to 20% at 34 μM (Figure 1A) and CPT 11 increased apoptosis from 9% at 5 μM to 16% at 15 μM (Figure 1B). These data suggest that apoptosis may have a role in drug-induced Caco-2 cells cytotoxicity especially at high concentrations of the drugs.

To evaluate whether aspirin may affect cell killing by topoisomerase drugs, we tested a schedule of administration in which the effects of aspirin treatment on Caco-2 cell growth were present at moment of anticancer-drug exposure: cells were pre-treated with aspirin for 24 h and subsequently exposed for 48 h to the aforementioned ranges of drugs in the continuous presence of NSAIDs (co-treatment). We evaluated the interference of 2 mM aspirin with anticancer drug induced apoptosis through the analysis of the sub-diploid DNA peak at flow cytometry. As shown in Figure 1, the levels of apoptosis were reduced for each concentration of both anticancer drugs when administered in the presence of aspirin compared with single agent treatment. Aspirin-co-treated Caco-2 cells were particularly resistant to etoposide-induced apoptosis: in fact the percentage of apoptosis in cells co-treated with aspirin and Vp-16 at 8.5 and 17 μM decreased from 14 to 7% and from 21 to 9%, respectively. The effect of aspirin on drug-induced apoptosis was similar when aspirin and anticancer drugs were simultaneously administered (data not shown). These results indicate that aspirin-dependent inhibition of proliferation is associated with a reduced ability of the drug to induce apoptosis.

**Figure 2:**
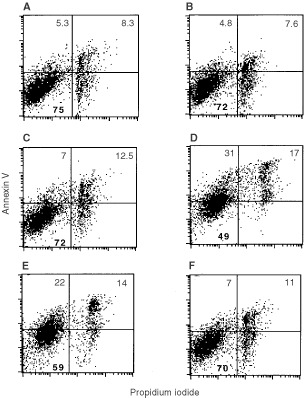
Effect of aspirin co-treatment on annexin V-FITC/PI staining of Caco-2 cells. Cells at day 3 were incubated with aspirin for 24 h, then anticancer drugs were added in presence of aspirin for 48 h. Four distinct phenotypes become distinguishable: (i) viable cells (lower left quadrant); (ii) cells at early stage of apoptosis (upper left quadrant); (iii) cells at late stage of apoptosis (upper right quadrant); (iiii) necrotic cells (lower right quadrant). (**A**) untreated cells; (**B**) cells treated with 2 mM Aspirin; (**C**) cells treated with 5 mM Aspirin; (**D**) cell treated with 17 μM Vp-16; (**E**) cells co-treated with 2 mM aspirin and 17 μM Vp-16; (**F**) cells co-treated with 5 mM aspirin and 17 μM Vp-16. Data represent one of three similar experiments.

To quantify the effects of aspirin co-treatment on Vp-16-induced apoptosis and to determine whether this effect was present at concentration in which aspirin was already apoptotic by itself, we used annexin V-FITC/propidium iodide staining assay. In fact this assay allows to clearly distinguish viable cells from those undergoing different stages of apoptosis or necrosis. The effect of aspirin at 2 and 5 mM concentration on Vp-16-induced toxicity in Caco-2 cells is analysed in Figure 2

**Figure 3:**
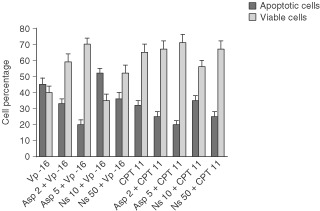
Effect of aspirin and NS-398 co-treatment on 17 μM Vp-16- and 10 μM CPT 11- induced apoptosis of Caco-2 cells. The percentage of viable and apoptotic cells were calculated as reported in Figure 2. Apoptotic bars are the sum of percentage of cells at early and late stages of apoptosis. Data are expressed as mean±s.d. Data points represent the mean of triplicate experiments.

. Dot plots of green (Annexin V-FITC) *vs* red (PI) fluorescence showed four separate clusters: viable cell (lower left quadrant), cells at early stage of apoptosis, (upper left quadrant), cell at late stage of apoptosis (upper right quadrant), necrotic cells (lower right quadrant). Because of the high sensitiveness of this procedure, we detected higher levels of apoptosis as compared to that obtained at the analysis of sub-diploid peak. Vp-16 at 17 μM increased the percentage of early and late apoptotic cells from 13 to 48% and decreased the percentage of viable cell from 75 to 49% (Figure 2A,D). Aspirin co-treatment at 2 and 5 mM dose-dependently decreased the percentage of early and apoptotic cells from 48 to 36 and 18%, respectively, (Figure 2D *vs* E,F) and increased the percentage of viable cells from 49 to 59 and 70% (Figure 2D *vs* E,F); similar data were obtained for Vp-16 at 8.5 μM (data not shown) and CPT 11 at 10 μM (Figure 3

). Not significant change in the levels of necrosis was observed.

We also evaluated whether the effects of aspirin co-treatment on anticancer drug responsiveness were unique of Caco-2 cells or were obtainable in other colon cancer cells showing different Cox isoform expression profile. We selected the colon cancer cell line SW480 being Cox-1 positive, but Cox-2 negative (Smith *et al*, 2000). SW480 cells were co-treated with aspirin and topoisomerase inhibitors under the above mentioned experimental conditions. Aspirin co-treatment at 2 and 5 mM decreased both CPT 11- and VP-16-induced apoptosis. The effect was dose-dependent and in the same range of magnitude of that obtained in Caco-2 cells (data not shown).

### Effect of NS-398 co-treatment on Vp-16 and CPT-11-induced apoptosis in Caco-2 cells

Data from our laboratory indicated that Caco-2 cells express Cox-2 but not Cox-1 and NS-398, a COX-2 selective inhibitor, inhibited DNA synthesis and induced apoptosis in Caco-2 cells (Di Popolo *et al*, 2000). To evaluate whether the effects of aspirin co-treatment on Caco-2 cells were dependent on the inhibition of Cox-2 activity, we analysed the effect of NS-398 co-treatment on anticancer drug-induced apoptosis.

**Figure 4:**
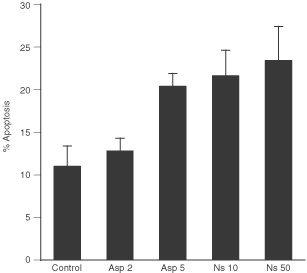
Effect of 2 and 5 mM aspirin and 10 and 50 μM NS-398 72 h treatment on apoptosis of Caco-2 cells. The percentage of Caco-2 cells at early and late stages of apoptosis was quantified as reported in Figure 2. Data points represent the mean of triplicate experiments±s.d.

We firstly evaluated the effect on apoptosis of NS-398 72 h treatment at different concentration using annexin V-FITC/propidium iodide staining assay. As shown in Figure 4

, 10 and 50 μM NS-398 treatment induced higher level of apoptosis compared with 2 and 5 mM aspirin treatments in Caco-2 cells (22 and 25% compared with 12 and 20%, respectively).

Then we evaluated the effect of NS-398 co-treatment on Vp-16- and CPT 11-induced toxicity. The percentage of cells in early plus late apoptosis *vs* viable cells induced by co-treatments with aspirin or NS-398 and Vp-16 or CPT 11 are given in Figure 3.

Aspirin at 2 and 5 mM dose-dependently decreased both Vp16- and CPT 11-induced apoptosis and increased cell viability. NS 398 at 10 μM increased both 17 μM Vp-16- and 10 μM CPT 11-induced apoptosis (*P*<0.05 and *P*>0.05, respectively), while NS 398 at 50 μM decreased 17 μM Vp-16- and 10 μM CPT 11-dependent apoptosis (*P*>0.05 and *P*<0.05, respectively). NS 398 at 10 μM did not significantly alter the percentage of viable cells following treatment with Vp-16- or CPT 11 co-treated cells, while NS 398 at 50 μM increased cell viability of Vp-16-treated cells from 40 to 52% (*P*<0.05). No effects were obtained on anticancer drug induced-apoptosis with NS 398 at 1 μM (data not shown).

### Effect of anticancer drug treatments and anticancer drug plus aspirin or NS-398 co-treatments on cell cycle distribution

We examined whether aspirin and NS-398 treatments had any distinct effect on cell cycle parameter under the same experimental conditions. The distribution of cells in the phases of the cell cycle is presented in Table 1

**Table 1:**
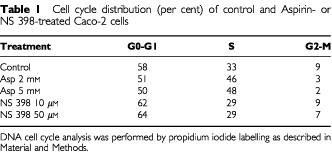
Cell cycle distribution (per cent) of control and Aspirin- or NS 398-treated Caco-2 cells

. Aspirin treatment was associated with a modest dose-dependent increase in the proportion of cells in the S phase, while NS-398 did not significantly alter cell cycle parameters with respect to control cells.

We also asked whether the different effect of aspirin and NS-398 on survival could be determined by a selective interference with anticancer drug-induced cell cycle arrest. In fact, Vp-16 arrests cells in the pre-mitotic phase of the cell cycle leading to accumulation of the cells in the late S or G2 phase (Fearnhead *et al*, 1994; Downes *et al*, 1994); while CPT 11 causes S phase slowing (Shao *et al*, 1997; Mcdonald and Brown, 1998). Effect of Vp-16 and CPT 11 treated, Vp-16 and CPT 11 plus aspirin or plus NS-398 co-treated cells under previously described experimental conditions are shown in Table 2

**Table 2:**
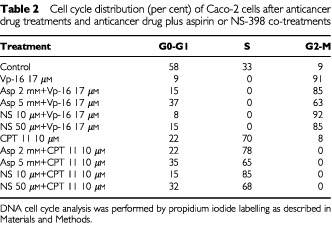
Cell cycle distribution (per cent) of Caco-2 cells after anticancer drug treatments and anticancer drug plus aspirin or NS-398 co-treatments

. We found that Vp-16, from lowest concentration of drug tested, increased the distribution of Caco-2 cells at the G2 phase of the cell cycle. In particular, VP-16 at 8.5 μM and 17 μM treatments were able to trap 85 and 91% of Caco-2 cells in the G2 phase of cell cycle, respectively. Whereas CPT 11 at 10 and 15 μM treatments accumulated 70 and 85% of Caco-2 cells in the S phase of cell cycle, respectively (Table 2 and data not shown). Aspirin and NS-398 co-treatment had different effect on anticancer drug-induced cell cycle distribution: in cells that have been co-treated with Vp-16 at 17 μM, aspirin dose-dependently increased the percentage of Caco-2 cells at G0–G1 phase of cell cycle. NS-398 at 10 μM had no effect on Vp-16-induced accumulation at the G2 phase of cell cycle, while at 50 μM increased the percentage of cells at G0–G1 phase from 9 to 15%. In cells that have been co-treated with aspirin and CPT 11 at 10 μM there was an increase in the percentage of cells at G0–G1 phase similar to that observed in Vp-16 co-treated cells. NS-398 only at 50 μM concentration increased the percentage of cells at G0–G1 phase from 22 to 32% (Table 2).

### Effect of aspirin co-treatment on Caco-2 plating efficiency

**Figure 5:**
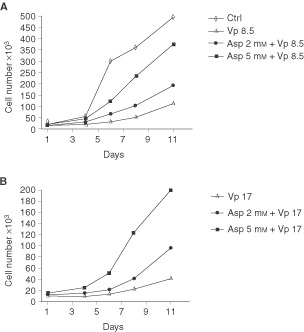
Effect of 2 and 5 mM aspirin and VP-16 at 8.5 μM (**A**) or 17 μM (**B**) co-treatments on plating efficiency of Caco-2 cells. After each treatment, 15 000 cells were seeded in triplicate in 24 multiwell dishes and counted at day 1, 4, 6, 8 and 11. All data points represent the mean of triplicate experiments.

Several reports indicate that cancer cells after a genotoxic treatment can take hours to many days before dying (Vidair *et al*, 1996; Han *et al*, 1997). To better evaluate the cytoprotective effect of aspirin, also with respect to potential delayed toxicity of Vp-16, we measured overall cell viability by performing a plating efficiency assay following 8.5 μM and 17 μM etoposide treatments and co-treatments with either 2 or 5 mM aspirin (Figure 5A,B

). All replated control Caco-2 cells started to divide after a lag period of 48h (Figure 5A); they entered into the exponential phase of cell growth at day 4, and reached the stationary phase at day 14 (data not shown). Etoposide treatments at both concentrations produced an approximately 0.7-fold decrease of the cell number at day 4 after replating; the fraction of surviving cells entered into the logarithmic phase of cell growth only at day 8. On the contrary, aspirin co-treated cells more efficiently replated and started the exponential phase of cell growth at day 6, 5 mM aspirin co-treated cells showing the best profile of cell growth. Thus, aspirin co-treated cells appeared to be more viable and less sensitive to anticancer delayed toxicity. Etoposide at 34 μM was such a toxic treatment that cell restarted to divide only at day 11; nevertheless, cells co-treated with aspirin and etoposide also at this concentration, continued to show an advantage in cell growth (data not shown).

### Effect of different treatments on the expression of the bcl-2 protein family

**Figure 6:**
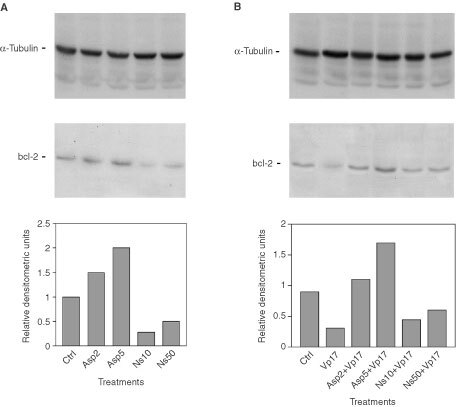
(**A**) Effect of single agent treatment on α-tubulin and bcl-2 expression in Caco-2 cells. Upper panel: Western immunoblot analysis of protein lysates from control cells (lane 1), from cell treated with aspirin for 72 h at 2 mM (lane 2) and 5 mM concentrations (lane 3), from cell treated with NS-398 for 72 h at 10 μM (lane 4) and 50 μM (lane 5). Lower panel: densitometric analysis of the autoradiograph shown. A representative experiment of three separate ones is shown. (**B**) Effect of aspirin and NS-398 co-treatments on α-tubulin and bcl-2 expression in Caco-2 cells. Upper panel: Western immunoblot analysis of protein lysates from untreated (lane 1*)*, from cell treated with VP-16 for 48 h at 17 μM (lane 2*),* from cell co-treated with 17 μM VP-16 and 2 mM aspirin (lane 3), from cells co-treated with 17 μM VP-16 and 5 mM aspirin (lane 4), from cells co-treated with 17 μM VP-16 and 10 μM NS-398 (lane 5), from cells co-treated with 17 μM VP-16 and 50 μM NS-398 (lane 6). Lower panel: densitometric analysis of the autoradiograph shown. A representative experiment of three separate ones is shown.

The proteins belonging to the Bcl-2 family are important regulators of cell death in eukaryotes (Yang *et al*, 1995; Korsmeyer, 1999). The apoptotic response of a cell damage by chemotherapy may depend at least in part, on the balance between proteins which predispose against genotoxic to programmed cell death such as bad and bax and proteins which antagonise programmed cell death, such as bcl-2 and bclx-xl (Yang *et al*, 1995; Korsmeyer, 1999). To study the mechanisms that mediate the effect of aspirin and NS-398 on anticancer drugs Caco-2 cell sensitivity, we investigated whether these treatments were associated with a modulation of the expression of these proteins. Soluble cell lysates were obtained from control cells and from cell treated with aspirin at 2 and 5 mM and NS-398 at 10 μM and 50 μM doses for 72 h. Expression of bad, bax and bclx-xl were not affected by any treatment (data not shown); in contrast, more extensive modifications were observed in the levels of bcl-2 (Figure 6

A): aspirin at 2 and 5 mM increased bcl-2 levels dose-dependently by 1.5- and two-fold, respectively, compared with untreated cells; on the contrary, NS-398 at 10 μM and 50 μM decreased the levels of bcl-2 by three- and two-fold, respectively. Thus, aspirin, but not NS-398, treatment increased the ratio bcl-2 to bax determining a pattern of survival in Caco-2 cells, which was most evident at 5 mM treatment. Then we asked whether etoposide treatment had any effect on bcl-2 levels: Vp-16 treatment alone decreased bcl-2 levels by three-fold compared with control cells (Figure 6A); on the contrary, the levels of bcl-2 were three-fold and five-fold increased in cells co-treated with Vp-16 and aspirin at 2 and 5 mM compared with Vp-16 treated cells, respectively (Figure 6B). bcl-2 levels were increased by approximately 1.5-fold and two-fold in Vp-16 co-treated cells with NS 398 at 10 and 50 μM, respectively, compared with Vp-16 treated cells (Figure 6B). The above all data indicated that the effects of aspirin and NS-398 treatments on bcl-2 protein family expression persisted in co-treatment and again were consistent with the effects of aspirin and anticancer drugs administrations at different doses on apoptosis of Caco-2 cells.

## DISCUSSION

Mounting evidence suggest that aspirin and other NSAID are able to interfere with mitogenic signalling causing arrest of cells in the G0/G1 phase of the cell cycle and, depending on the doses and the drug, to induce apoptosis as conventional cytotoxic drugs (Lu *et al*, 1995; Shiff *et al*, 1995, 1996; Piazza *et al*, 1995; Arber *et al*, 1997). For these properties NSAIDs have been sporadically employed in clinical cancer therapy; more frequently, they are utilised as analgesic, occasionally concurrently chemotherapy administration without a profound knowledge of the potential interaction with anticancer drugs and their efficacy. To date, very little information has been reported on this subject; a paper describes the first extensive screen of commercially available NSAIDs with anticancer drugs and discusses the potential clinical benefits of such combinations (Duffy *et al*, 1998). More recently, several studies have investigated the possibility of Cox-2 selective inhibitor-mediated enhancement of chemotherapeutic drug toxicity (Soriano *et al*, 1999; Hida *et al*, 2000). There are not reports comparing the effects of Cox-2 selective inhibition on responsiveness of colon cancer cells to chemotherapy with that obtained with aspirin, the most frequently used medicinal drug also for its anti-aggregating properties and in the clinical treatment of inflammatory diseases. In particular, the use of aspirin for analgesia and antipyresis (650 mg of oral administration, six times more on average than the anti-platelet dose) results in peak plasma concentration of 25 μg ml (which corresponds approximately to 0.15 mM). On the other hand, the high dose of aspirin required for the treatment of arthritis (4–6 g of oral administration) determines aspirin plasma level up to 300 μg ml, which corresponds to 2 mM (Insel, 2001). The main purpose of this study is to evaluate if combined treatment *in vitro* would produce interactive effects that could be relevant in the clinical use. We have previously demonstrated that aspirin and NS-398 treatments are both associated with the inhibition of proliferation and induction of apoptosis of Caco-2 cells (Ricchi *et al*, 1997; Di Popolo *et al*, 2000). Numerous studies suggest that any interference with mitogenic signalling and cell cycle cause a modification in the responsiveness to conventional cytotoxic drugs. In the present study, we investigate whether aspirin and NS-398 interference with cell proliferation has any effect on Caco-2 cells responsiveness to the topoisomerase poisons irinotecan and etoposide, anticancer agents that specifically require DNA synthesis to exert their toxicity.

The data reported herein demonstrate that aspirin and NS-398 act as anticancer agents in Caco-2 colon cancer cells with cytostatic properties and with different ability to induce apoptosis at high dosages. Also, we show that they differently modify anticancer responsiveness to chemotherapy of Caco-2 cells. We have evaluated this effect mainly towards the induction of apoptosis. In fact, there is emerging evidence that resistance to antitumour treatments relies on reduced sensitivity to apoptosis induction (Fisher, 1994; Nagata, 1997); in this regard, our results demonstrate that aspirin treatment is able to counteract the proapoptotic effect of anticancer drugs *in vitro*.

Aspirin co-treatment produces, at all concentrations investigated, a reduction of anticancer drug-induced apoptosis in Caco-2 cells and, at 5 mM, a reduction of S/G2 phase cell cycle accumulation. We hypothesise that the effects of high doses of aspirin co-treatment on anticancer drug-induced toxicity could be mainly explained by the almost complete inhibition of DNA synthesis and G1 cell cycle arrest obtained at this dose of aspirin that have been shown also to alter cell cycle-related proteins (Law *et al*, 2000; Marra *et al*, 2000); the reduced ability of such anticancer drugs to induce apoptosis in quiescent cells have been already observed (Nitiss and Wang, 1996: Lin *et al*, 1998). Furthermore, it is a common observation that agents able to interfere with cell cycle usually prevent the action of drugs active in the next phase of cell cycle (Lin *et al*, 1998).

Moreover, we clearly demonstrate by PI- and annexin V-staining that the reduced levels of apoptosis obtained in aspirin co-treatments are associated with an increase in cell viability of Caco-2 cells; this effect is also evident by the analysis of plating efficiency in aspirin-Vp 16 co-treated cells; thus, these data indicate that aspirin co-treatment interferes not only with apoptotic death but also with overall toxicity induced by these anticancer drugs. In this regard, aspirin and its metabolite sodium salicylate have been found to be protective against neurotoxicity elicited by the excitatory amino acid glutamate in rat primary neuronal cultures (Grilli *et al*, 1996); thus, our data may represent a further evidence of aspirin cytoprotective property against a cellular damage.

In addition, PI- and annexin V-staining assay indicated that the cytoptotective effect of aspirin co-treatment is present not only in Caco-2 cells but also in the Cox-2 negative, Cox-1 positive Sw 480 colon cancer cell line. These data may suggest that aspirin interferes with topoisomerase poison-induced toxicity through a Cox-independent mechanism.

On the other hand, our data show that NS-398 co-treatment may alternatively reduce or increase anticancer drug induced apopotosis, depending on the concentration. These effects are less powerful compared with those observed in the presence of aspirin. NS-398 at 1μm concentration completely inhibits the biosynthesis of prostaglandin E_2_ in Caco-2 cells (Acquaviva *et al*, unpublished data) without affecting anticancer drug responsiveness; NS 398, at 10 μm concentration, causes minimal enhancement of anticancer drug-induced apoptosis without modifying cell cycle parameters. On the contrary, our data show that NS-398, at dose of 50 μM, is cytoprotective and counteracts anticancer drugs-induced cell cycle perturbation. Based on these findings, we postulated that the described effects of increasing dose of NS-398, as in the case of aspirin, might be dependent on regulation of cell cycle regulatory protein (Hung *et al*, 2000) and not related on the inhibition of Cox-2 activity; these data could also reinforce the hypothesis of the concurrent presence of Cox-2-dependent and independent mechanisms of action in the antiproliferative effect of individual selective Cox-2 inhibitors (Grösch *et al*, 2001; Smith *et al*, 2000).

Several reports suggest that the regulation of the cell response to chemotherapeutic drugs may also involve a dynamic interplay among the bcl-2 protein family members (Yang and Korsmeyer, 1996), on which novel treatment approaches have focused to overcame drug resistance (Strobel *et al*, 1998a,b; Kobayashi *et al*, 1998). In this regard, searching for an alternative and/or complementary mechanism to cell cycle block, we have evaluated the effect of aspirin and NS-398 treatment on these proteins. Levels of bax, bad and bclx-xl are not significantly affected by these treatments. On the contrary, we demonstrate that aspirin treatment is associated with a dose dependent increase of bcl-2, that play a pivotal role extending cell survival through the inhibition of apoptosis induced by various stimuli, including chemotherapeutic drugs (glucocorticoids, alkylating agents, topoisomerase II inhibitors). The increase in bcl-2 levels is more evident at dose of 5 mM aspirin, is detectable in co-treatment and may be responsible for the induction of a survival signal in Caco-2 cells and for the increase in viability observed in co-treatments. It has been recently demonstrated that bcl-2 expression is increased at G1 phase of cell cycle (Gao and Dou, 2000). Because we have demonstrated that 5 mM aspirin treatment in the presence of Vp-16 and CPT 11 increases the percentage of cells at G1 phase, we postulated that the observed increase of bcl-2 expression might be dependent on a G1 phase arrest. However, 5 mM aspirin treatment determines a modest increase in the levels of apoptosis also in the presence of increased bcl-2 expression; further studies are needed to clarify this phenomenon. Moreover, the levels of antiapoptotic bcl-2 protein are decreased by NS-398 treatment at 10 μM and to a lesser extent at 50 μM. These modifications persist after anticancer drug treatment and again strictly correlate with data on apoptosis in co-treatments; these findings confirm previous reports showing that bcl-2 protein levels can affect apoptosis induced by etoposide (Kamesaki *et al*, 1993; Dole *et al*, 1994). Further studies are needed to investigate if the effects we observed *in vitro* are specific for a cancer cell type and for phase specific anticancer agents or are a more general phenomenon reproducible *in vivo* also for the aspirin metabolite salicylate. In fact, preliminary reports indicate that this aspirin metabolite also affects cell growth (Elder *et al*, 1996).

Finally our data prompt to accurately investigate all the possible interference between aspirin or others NSAID and anticancer drugs and other genotoxic damages *in vivo* and *in vitro*. Our finding may have important implications for treatment schedules involving both cytotoxic agents and aspirin in malignancies; they could suggest to absolutely avoid co-treatment of aspirin and anticancer drugs. On the other hand, they could also provide a rationale for novel strategies of enhancement of chemotherapy activity: in fact, Cox-2 selective inhibition could be a feasible and not toxic tool to increase chemotherapy effectiveness.
